# Antigen Presenting Cells from Tumor and Colon of Colorectal Cancer Patients Are Distinct in Activation and Functional Status, but Comparably Responsive to Activated T Cells

**DOI:** 10.3390/cancers13205247

**Published:** 2021-10-19

**Authors:** Frank Liang, Azar Rezapour, Louis Szeponik, Samuel Alsén, Yvonne Wettergren, Elinor Bexe Lindskog, Marianne Quiding-Järbrink, Ulf Yrlid

**Affiliations:** 1 Department of Microbiology and Immunology, Institute of Biomedicine, Sahlgrenska Academy, University of Gothenburg, 405 30 Gothenburg, Sweden; azar.rezapour@gu.se (A.R.); louis.szeponik@gu.se (L.S.); samuel.alsen@gu.se (S.A.); marianne.quiding-jarbrink@microbio.gu.se (M.Q.-J.); 2 Department of Surgery, Institute of Clinical Sciences, Sahlgrenska University Hospital, University of Gothenburg, 413 45 Gothenburg, Sweden; yvonne.wettergren@gu.se (Y.W.); elinor.bexe-lindskog@surgery.gu.se (E.B.L.)

**Keywords:** colorectal cancer, tumor microenvironment, antigen presenting cells, T cells

## Abstract

**Simple Summary:**

Colorectal cancer (CRC) remains the third most common cancer. Associations between intratumoral T cells, also known as tumor infiltrating lymphocytes (TILs), and the CRC patients’ responses to treatment have been described. Traditionally, TILs and antigen presenting cells (APCs) are studied separately on preserved CRC biopsies, disregarding the adjacent colonic tissue that would also be exposed to the administrated chemotherapy or radiotherapy. Thus, combined data sets on the subset composite and functional capacity of APCs and T cells within the same tumor, as well as colonic tissue, remain infrequent. Our phenotypic and functional comparison of T cell and APC subsets in tumor vs. colon from patients with CRC may give further insights into their propensity to maintain CRC treatment-induced immune responses locally in tumor and off-target colonic tissue.

**Abstract:**

Although mouse models of CRC treatments have demonstrated robust immune activation, it remains unclear to what extent CRC patients’ APCs and TILs interact to fuel or quench treatment-induced immune responses. Our ex vivo characterization of tumor and adjacent colon cell suspensions suggest that contrasting environments in these tissues promoted inversed expression of T cell co-stimulatory CD80, and co-inhibitory programmed death (PD)-ligand1 (PD-L1) on intratumoral vs. colonic APCs. While putative tumor-specific CD103+CD39+CD8+ TILs expressed lower CD69 (early activation marker) and higher PD-1 (extended activation/exhaustion marker) than colonic counterparts, the latter had instead higher CD69 and lower PD-1 levels. Functional comparisons showed that intratumoral APCs were inferior to colonic APCs regarding protein uptake and upregulation of CD80 and PD-L1 after protein degradation. Our attempt to model CRC treatment-induced T cell activation in vitro showed less interferon (IFN)-γ production by TILs than colonic T cells. In this model, we also measured APCs’ CD80 and PD-L1 expression in response to activated co-residing T cells. These markers were comparable in the two tissues, despite higher IFN- γ exposure for colonic APCs. Thus, APCs within distinct intratumoral and colonic milieus showed different activation and functional status, but were similarly responsive to signals from induced T cell activation.

## 1. Introduction

Cancer-related mortality remains high in patients with CRC [1,2]. It is widely accepted that TILs have prognostic value in CRC [3,4,5], and this has prompted the phenotypic definition of tumor-specific TILs. Previous studies have proposed the co-expression of CD103 and CD39 as markers for tumor-specificity on CD8+ TILs [6,7]. These memory T cells infiltrating the tumor likely interact with intratumoral APCs comprised of macrophages (MPs) and dendritic cells (DCs). The MPs are commonly divided into M1 (anti-tumor) and M2 (pro-tumor) subsets, although such distinction seems oversimplified due to the heterogeneity and plasticity of MPs [8,9]. Pro- or anti-tumor dichotomy has also been applied to DCs, since conventional DC 1(CDC1) may cross-present tumor antigens to CD8 T cells with tumor-killing potential, and CDC2 stimulate CD4 T cells that could promote or impede anti-tumor responses [10,11]. During APC-T cell interaction, MPs and DCs have the capacity to express cytokines or cell membrane-associated ligands that activate or inhibit effector functions of the interacting T cell. However, the role of DCs and MPs in CRC prognosis remains inconclusive [12,13,14,15]. 

Emerging reports associate TILs with clinical response to CRC treatments since treatment-induced immune activation indicated by the increase in TIL numbers was coupled with improved survival [16,17,18,19]. To date, immune checkpoint inhibition (ICI) with antibodies targeting PD-1 is approved for a subset of CRC patients. Although anti-PD-1 antibodies directly target T cells, models of ICI have demonstrated the prerequisite of specific DCs for tumor rejection [11,20,21]. Interestingly, the absence of co-stimulation is required for complete PD-1-mediated T cell inhibition [22]. Thus, to block inhibition conveyed by PD-1, mice treated with anti-PD-1 antibodies required specific T cell co-stimulation for efficient tumor elimination [23]. Since activated APCs highly express co-stimulatory molecules, they likely facilitate the unleashing of T cells in PD-1-targeting ICI. Further, mechanistic mouse models have also shown that radiotherapy indirectly augments T cell immunity, as initial APC activation by danger-associated molecules released from irradiated tumors were essential for tumor rejection [24,25]. Chemotherapy has also been reported to induce extracellular danger-associated molecules that activate APCs [26]. Altogether, potent and durable CRC treatment-induced intratumoral immune responses might depend on APC-T cell interactions and their mutual regulation.

Chemoradiation or ICI induce systemic immune responses [27,28], which involve off-target sites such as the tumor-adjacent colon. Here, we phenotypically and functionally characterized the APCs and T cells co-residing in the same tumor from CRC patients, as well as autologous colon tissue to ultimately address the responses of APCs exposed to induced activation of neighboring T cells.

First, we determined the ex vivo composition and activation status of T cell and APC subsets. We analyzed the expression of co-stimulatory CD80, and co-inhibitory PD-L1 on CD11c+CD64+CD14+ MPs and CD11c+CD64- DCs, plus subsets of the latter (i.e., CD141+ CDC1 and CD1c+ CDC2). We also evaluated the activation markers, CD69 and PD-1 on T cell subsets defined by CD103 and CD39 expression. Our concurrent assessment of surface activation markers enabled correlation analyses of APC and T cell subsets in the autologous tumor and colon to address potential relationships in their ex vivo activation status. Secondly, protein processing of APCs and T cell cytokine responses were determined in both tumor and colonic tissues. Lastly, we attempted to model general T cell activation potentially induced by direct or indirect T cell-targeting treatments via in vitro polyclonal T cell stimulation. We used this model to determine the regulation of CD80 and PD-L1 expression on tissue APCs by activated T cells.

Overall, our data obtained from a total of fifty-five CRC patients could provide further insight on whether intratumoral and colonic APCs exposed to activated T cells promote or limit the CRC treatment-induced immunological landscape.

## 2. Results

### 2.1. APCs in Both Tumor and Adjacent Colon Are Predominantly CD64+CD14+ MPs

**Figure 1:**
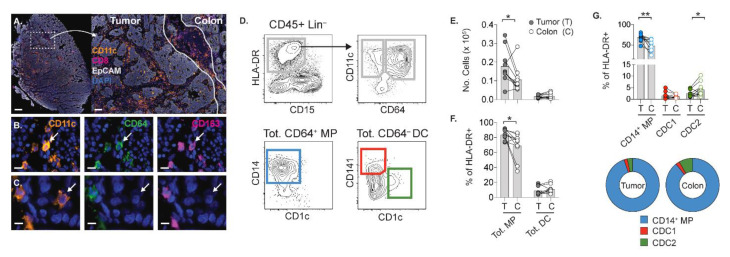
Characterization of APCs in tumor and adjacent colon from patients with CRC. (**A**) Tumor section showing CD11c+ APCs, CD8+ TILs and EpCAM+ epithelial cells. Arrows denote CD11c+CD64+CD163+ MPs (**B**) and CD11c+ CD64-CD163- DCs (**C**). Cell nuclei are DAPI+. Scale bars: (**A**) 500 μm (left) and 100 μm (right), (**B**) 10 μm and (**C**) 5 μm. (**D**) Gating strategy on tumor suspension showing CD45+ lineage (CD3/CD19/CD56)- HLA–DR+CD15-CD11c+ APCs divided into total CD64+ MPs containing CD14+ MPs (lower left panel), and total CD64- DCs comprising CD141+ CDC1 and CD1c+ CDC2 (lower right panel). Total MPs and DCs in tumor (T) and colon (C) by numbers per 1 million cells (**E**), or percent of HLA–DR+ APCs (**F**). (**G**) Percent and proportion of APC subsets within HLA–DR+ APCs. Bars show the mean. Connecting lines indicate autologous samples. (* *p* < 0.05, ** *p* < 0.01, Wilcoxon test).

Intratumoral APCs are instrumental in stimulation of anti-tumor responses of TILs [10,29]. Hence, we initiated our APC analyses by immunofluorescent staining of histological sections from the first of three patient cohorts included in the current study. The CD11c+ APCs were mainly found in the EpCAM- tumor stroma alongside CD8+ TILs (Figure 1A). Within the CD11c+ APCs, MPs were distinguished from DCs by their co-expression of CD64 and CD163 (Figure 1B), as these markers were absent on DCs (Figure 1C). In general, CD64 and CD163 plus an array of other markers are used for defining anti-tumor and pro-tumor MPs, respectively. However, expression of CD64 or CD163 is not always mutually exclusive since CD64+CD163+ MPs have been reported in CRC patients [9,30], which likely reflect the plasticity or heterogeneity of MPs. 

In the second cohort, frequencies and phenotypic activation of APCs were determined in cell suspensions of tumor, adjacent colon and peripheral blood mononuclear cells (PBMCs). In line with our in situ analyses, the lineage- HLA-DR+ CD11c+ APCs among total CD45+ leukocytes in tumor suspension comprised of CD64+ MPs and CD64- DCs, which, hereafter, are referred to as MPs and DCs, respectively (Figure 1D). The majority of MPs co-expressed CD14, and DCs contained the CD141+ CDC1 and CD1c+ CDC2 subsets. Relative to colon, MPs were significantly more prominent in the tumor (Figure 1E,F), as previously described [9,31]. A similar elevation in frequency or number of intratumoral DCs was not observed. At the subset level, CD14+ MPs remained elevated in the tumor, and the percentage of CDC2 was significantly decreased in the tumor, along with a slight increase in CDC1 (Figure 1G). Both tumor and colon had a large proportion of CD14+ MPs within the HLA-DR+ APCs, and CDC2 were proportionally higher than CDC1 in colon (Figure 1G). Collectively, these analyses show alterations in the composition of APCs subsets in tumors compared to the adjacent colon.

### 2.2. The Level of MPs Relative to TILs in MSS Tumors Exceeds That of MSI-H Tumors

Tumors with high microsatellite instability (MSI-H) generally display immune activation represented by, e.g., increased T cell infiltration [32,33]. We found no apparent correlation between the frequencies of MPs vs. DCs among the leukocytes in microsatellite stable (MSS) and MSI-H tumors (Supplementary Figure S1A). There were no correlations between tumor stage and the frequency of TILs and APCs (Supplementary Figure S1B). 

As infiltration of immune cells may indicate vigorous immune responses, we assessed whether frequencies of total CD3+ TILs associated with levels of MPs or DCs. We observed an inversed correlation between frequencies of TILs and MPs (Supplementary Figure S1C). However, percentages of DCs and TILs in MSI-H tumors displayed a trend towards a positive association, which was not statistically significant. Regarding the APC to TIL ratio, a significantly higher MP to TIL ratio was found in MSS tumors, which also surpassed the DC to TIL ratio in the same tumors (Supplementary Figure S1D). The ratios of the combined levels of MPs and DCs relative to TILs were also higher in MSS tumors, which could be due to, e.g., lower infiltration, survival and/or proliferation of T cells in these tumors. 

So far, T cell infiltration was assessed by the pan-T cell marker CD3 in cell suspensions for a general overview on APC and T cell frequencies. We revisited the cryopreserved tumors in the previous cohort to address the intratumoral location and levels of CD4 vs. CD8 TILs. The mean in situ numbers of CD3+ CD4 or CD8 TILs in MSS tumor centers tended to be lower than in MSI-H tumors (Supplementary Figure S1E), which may explain the higher APC to TIL ratio in MSS tumor suspensions from the second cohort. Combined numbers of CD4 and CD8 TILs in the tumor center or stroma were rather similar, regardless of MSI status. Despite the presence of more TILs in the center of some of the MSI-H tumors, increased mutational burden in our patient cohort was not related to increased levels of TILs or APCs.

### 2.3. MPs in Tumor and Colon Inversely Express Co-Stimulatory CD80 and Co-Inhibitory PD-L1

**Figure 2:**
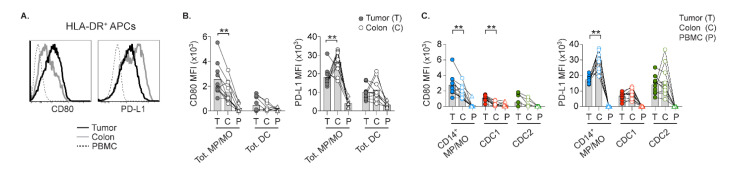
Expression of CD80 and PD-L1 on intratumoral and colonic APCs. (**A**) Histograms representing staining of CD80 or PD-L1 on total APCs in Tumor (T), Colon (C), and PBMCs (P). Mean fluorescence intensity (MFI) of CD80 and PD-L1 on total MPs or monocytes (MOs) and DCs (**B**), or on CD14+MPs/MOs, CDC1 and CDC2 (**C**). Bars show the mean. Connecting lines indicate autologous samples. (** *p* < 0.01, Wilcoxon test).

The abundance of TILs in the tumor stroma (Supplementary Figure S1E), where the majority of APCs also reside (Figure 1A–C), would enable frequent APC-T cell interactions. We, therefore, addressed the expression level of co-stimulatory CD80 and co-inhibitory PD-L1 by APCs, since these ligands have opposed effects on T cell activation (Figure 2A). As the microenvironment impacts APC-T cell interactions, we also included analyses of the adjacent colon and autologous PBMCs. 

While intratumoral MPs had higher CD80 levels than colonic MPs, the latter also expressed higher PD-L1 (Figure 2B). This inversed CD80 and PD-L1 expression was not equally apparent on the DCs. As CD14+ MPs were the predominant MP subset (Figure 1D), their inversed pattern of CD80 and PD-L1 expression in tumor and colon remained unchanged (Figure 2C). The expression of CD80, but not PD-L1, was significantly upregulated on intratumoral CDC1. However, CDC2 in both tumor and colon had diverse expression of these markers. In contrast to tissue APCs, monocytes and DCs in PBMCs hardly expressed CD80 or PD-L1. The expression pattern of CD80 and PD-L1 in tumor and colon suggests that APCs within these discrete tissue environments have discrepancies in their capacity to promote and limit T cell activation.

### 2.4. Tissue-Resident Memory T Cells in Tumor and Colon Express Opposed PD-1 and CD69 Levels

**Figure 3:**
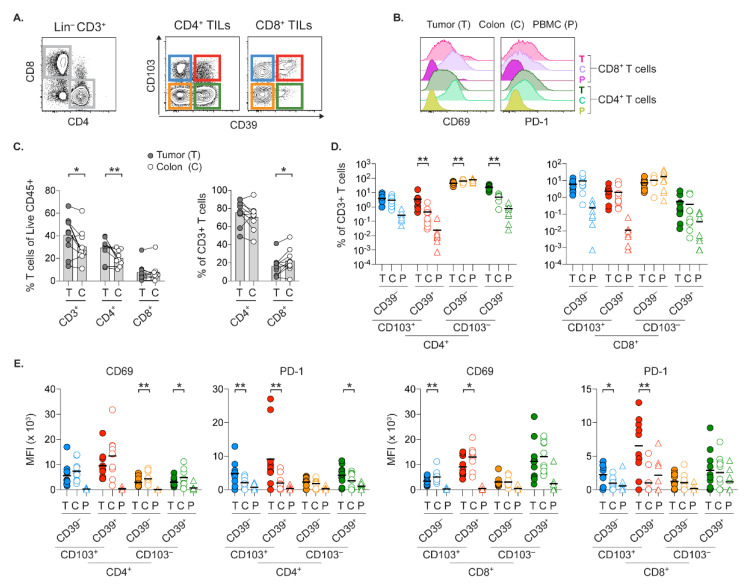
Phenotypic assessment of TILs and colonic T cells. (**A**) Gating strategy on tumor suspension where (CD11c/CD15/CD19)- CD3+ TILs are separated into CD4+ vs. CD8+ T cells and from which, four T cell subsets are subsequently defined by CD103 and CD39 expression. (**B**) Histogram overlays of CD69 and PD-1 staining on CD4 vs. CD8 T cells at specified sites. (**C**) Percent of total CD3+ T cells and CD4 or CD8 T cells within indicated population. (**D**) Percent of T cell subsets defined by CD103 and CD39 within CD3+ T cells. (**E**) MFI of CD69 and PD-1 staining on indicated T cell subsets and sites. Bars and bold horizontal line show the mean. Connecting lines indicate autologous samples. (* *p* < 0.05, ** *p* < 0.01, Wilcoxon test).

Additional regulation of tissue APCs’ CD80 and PD-L1 expression could stem from effector molecules from neighboring memory T cells acting on APCs. In this regard, the previously reported CD8+CD39+CD103+ tumor-specific TILs [6,7] could potentially be instrumental in regulation of these APC markers during tumor antigen-specific restimulation. However, CD39 or CD103 expression are also assessed in other aspects. For example, CD39 identifies regulatory T cells (Tregs) [34], and CD103 indicates tissue retention on tissue-resident memory T cells (TRMs) [35]. To this end, whether the frequency of CD4 or CD8 T cell subsets solely defined by CD103 and CD39 expression differ at several autologous sites with distinct milieus is largely unexplored. Thus, we assessed these T cell subsets in the second patient cohort (Figure 3A), from which we had access to matched tumor, colon and PBMC samples.

Initial T cell characterization showed higher levels of CD69 and PD-1 on CD4 and CD8 T cells in tissues, compared to PBMCs (Figure 3B). The early T cell activation marker CD69, also identifies TRMs at e.g. mucosal sites and in certain non-CRC tumors [35,36]. Thus, the wide range of CD69 levels on TILs, and more uniform CD69 expression on colonic T cells could represent varying degrees of tissue retention and/or T cell activation. Further, PD-1 upregulation by T cells denotes extended activation with or without exhaustion. Relative to the colon, frequencies of CD3+ TILs and CD4+ TILs within leukocytes were increased in the tumor (Figure 3C). Conversely, the percentage of CD8+ T cells among total T cells were slightly lower in the tumor compared to adjacent colonic tissue.

The frequency of CD4+CD39+ TILs, which likely contain the Tregs were significantly higher in the tumor regardless of their CD103 expression (Figure 3D). Both tumor and colon had similar levels of CD103+CD39- CD4 or CD8 TRMs. As expected, circulating T cells in PBMCs had low CD103 expression. The CD103- T cells in tissues might represent T cells that have yet to establish tissue residency and/or those about to exit the tissue. It is unclear if CD103 and CD39 on colonic CD8 T cells correspond to specificity for colonic antigens as co-expression of these markers on CD8 TILs suggest tumor specificity [6,7]. Relative to the colon, the frequency of potentially tumor-specific CD8+CD103+CD39+ TILs was not increased (Figure 3D). However, these TILs had significantly higher PD-1 and lower CD69 expression compared to their counterparts in colon (Figure 3E). In fact, the pattern of elevated PD-1 and decreased CD69 was observed on CD103+ CD4 or CD8 TILs, linking tumor residency to specific degree of TIL activation status. 

### 2.5. Association of Activation Status between Particular APCs and T cells Are Tissue-Specific

**Figure 4:**
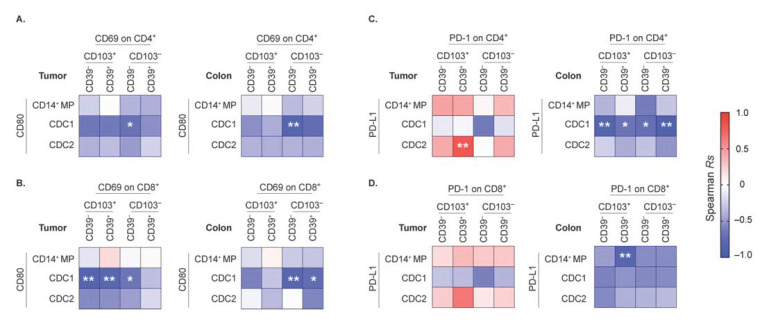
Correlation analyses of ex vivo surface activation markers of APC and T cell subsets. Heat maps from Spearman correlation between CD80 MFI of APC subsets vs. CD69 MFI of CD4 (**A**), or CD8 (**B**) T cell subsets in tumor and adjacent colon. Similar analyses on PD-L1 MFI of APC subsets vs. PD-1 MFI on CD4+ (**C**), or CD8+ (**D**) T cell subsets. (* *p* < 0.05, ** *p* < 0.01). Spearman *Rs* values and *p*-values are specified in Supplementary Figure S2A–D.

Our simultaneous characterization of multiple co-residing APC and T cell subsets encompassed the relatively rare opportunity to address whether the activation status of these cell subsets could be correlated (Figure 4A–D and Supplementary Figure S2A–D). In this regard, the opposed expression of PD-1 and CD69 on specific T cells (Figure 3E), as well as CD80 and PD-L1 on APCs (Figure 2A) in tumor vs. colon tissue, suggest discrete activation patterns in these tissues. 

Correlation analyses showed that associations between CD80 on APC subsets vs. CD69 on T cell subsets defined by CD103 and CD39 were generally negative in the tumor and colon (Figure 4A,B). In contrast, PD-L1 on intratumoral CD14+ MPs tended to be positively linked to PD-1 expression on CD4 and CD8 TIL subsets (Figure 4C,D), but an opposite trend was observed in the colon. This may suggest that MPs in the tumor support PD-1-mediated T cell inhibition, but colonic MPs prefer to reduce signals conveyed by PD-1. 

Higher PD-L1 levels on CDC1 in both tumor and colon were linked to lower PD-1 expression on T cell subsets (Figure 4C,D). In contrast, PD-L1 on intratumoral CDC2 was especially associated with increased PD-1 expression on CD4+CD103+CD39+ TILs containing the Tregs (Figure 4C). Furthermore, elevated CD80 levels on intratumoral CDC1 were highly associated with lower CD69 expression on CD103+ CD8 TILs (Figure 4B), but conversely, the association was more pronounced with CD103- CD8 T cells in the colon. This may indicate that DCs are interacting with different T cell subsets in these two tissues. For example, activated CDC1 preferentially promote CD69 downregulation on CD8+ TRMs in the tumor, whereas in the colon, the CDC1 mediate this effect on CD8+ former TRMs or non-TRMs.

### 2.6. APCs in Tumor and Colon Display Opposed Co-Stimulatory Capacity upon Protein Digestion

**Figure 5:**
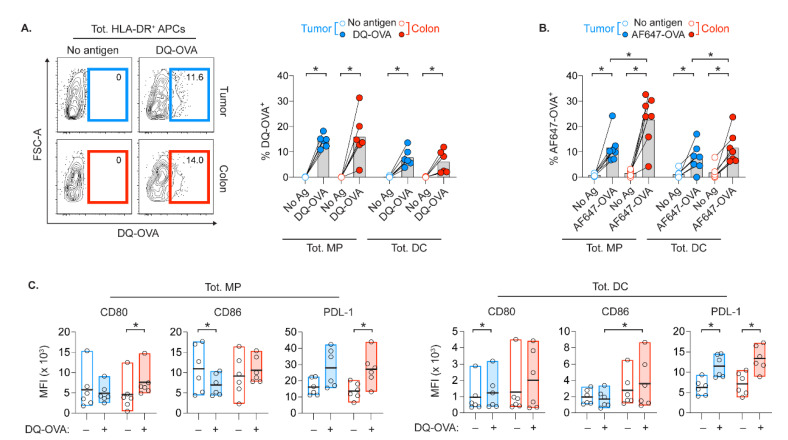
Protein uptake and degradation capacity of APCs from tumor and colon. (**A**) Flow cytometry plots on HLA-DR+ APCs with values indicating percent of APCs that degraded DQ-OVA protein (i.e., DQ-OVA+), and compiled data on DQ-OVA+ MPs and DCs. (**B**) Percent of MPs and DCs that ingested and/or degraded AF647-conjugated OVA. (**C**) Comparison of CD80, CD86 and PD-L1 MFIs on MPs and DCs in DQ-OVA–cultures that degraded DQ-OVA (DQ-OVA+), or not (DQ-OVA–). Bars and bold line in min-max bars show the mean. Connecting lines show control and treated cell suspension from the same patients. (* *p* < 0.05, Wilcoxon test).

Next, we assessed the functional capacity of APCs from the distinct environments of the colon and tumor. The expression of co-stimulatory and co-inhibitory markers on APC subsets from these tissues likely impact T cell stimulation during antigen presentation. Thus, we determined the APCs’ capacity to take up and degrade the protein antigen ovalbumin (OVA), and their subsequent expression of activation or inhibitory markers. In the presence of low concentration of OVA that only becomes fluorescent upon degradation (DQ-OVA), there were similar frequencies of DQ-OVA+ APCs in cell suspensions of both colon and tumor (Figure 5A). However, when evaluating both OVA uptake and degradation using low amounts of AF647-labeled OVA, the colonic APCs were more efficient (Figure 5B).

We also assessed whether protein degradation would subsequently enhance the APCs’ capacity to stimulate T cells. Comparison of DQ-OVA+ and DQ-OVA- APCs within the same cultures receiving DQ-OVA allows analyses of co-stimulatory CD80, CD86 and co-inhibitory PD-L1 on APCs that degraded OVA vs. those that were less efficient. Relative to internal DQ-OVA- control, there was a significant downregulation of CD86 on DQ-OVA+ MPs in the tumor, while upregulation of CD80 was observed on DQ-OVA+ colonic MPs (Figure 5C). Colonic DQ-OVA+ DCs upregulated CD86 at significantly higher levels than DQ-OVA+ DCs from the tumors. Interestingly, APCs in both tissues upregulated PD-L1 after OVA degradation. In conclusion, OVA degradation efficiency of APCs was generally not impacted by their tissue-specific stimulatory and inhibitory states. However, APCs from tumor vs. colon had distinct levels of co-stimulatory markers following OVA digestion. 

### 2.7. APCs Co-Express CD80 and PD-L1 in Presence of Activated T Cells In Vitro

**Figure 6:**
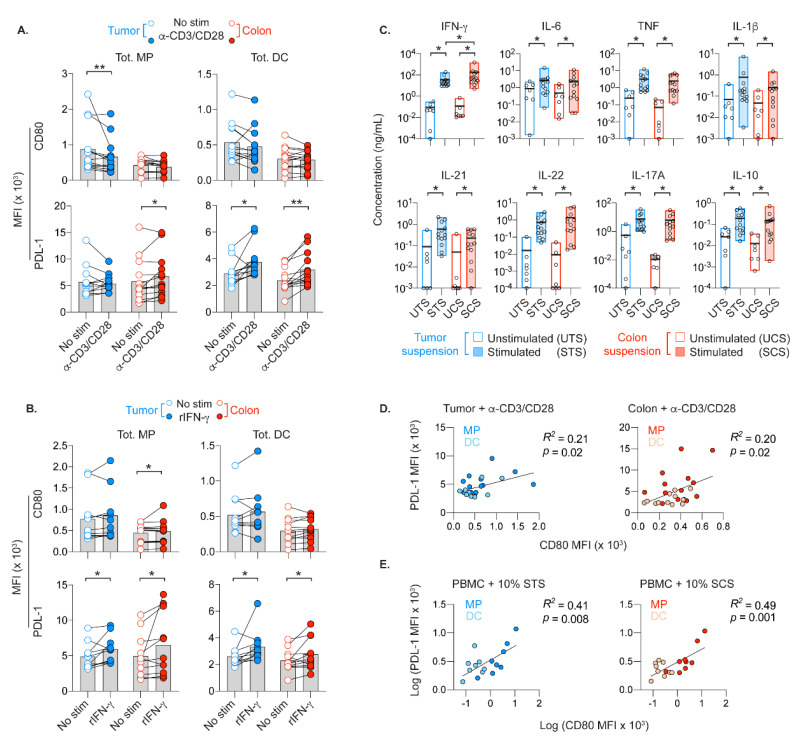
CD80 and PD–L1 expression on APCs in the presence of induced T cell activation. CD80 and PD–L1 MFI on MPs and DCs in tumor or colon suspensions after 6 h culture in media alone, or with microbeads loaded with anti-CD2/CD3/CD28 antibodies (referred to as microbeads) (**A**), or recombinant IFN-γ (**B**). (**C**) Cytokine levels after 6 h of culture. UTS and STS denote supernatants from unstimulated and microbeads-stimulated tumor suspensions, respectively. Likewise, supernatants from colon suspensions are referred to as UCS and SCS. Linear regression analyses of CD80 vs. PD–L1 MFI of APCs in microbeads-stimulated tissue suspensions (**D**), or in PBMCs exposed to 10% STS or SCS (**E**). Bars and bold line in min–max bars show the mean. Connecting lines show control and treated cell suspensions from the same patients (* *p* < 0.05, ** *p* < 0.01, Wilcoxon test).

APCs providing T cell stimulation was vital for tumor rejection in mouse models of CRC treatments [23,24,25]. We, therefore, determined the expression of CD80 and PD-L1 on APCs in our attempts to model treatment-induced T cell activation in vitro. As ICI and chemoradiation directly or indirectly target T cells regardless of their specificity, we simulated global T cell activation using microbeads loaded with anti-CD2/CD3/CD28 antibodies (referred to as microbeads) in the third CRC patient cohort (Figure 6A–D). Since off-target tissues are also exposed to CRC treatments, we included microbead-stimulated colonic suspensions to our analyses. 

In the midst of T cells activated by microbeads, downregulation of CD80 was observed only on intratumoral MPs, while DCs in tumor and colon retained their CD80 levels (Figure 6A). The colonic MPs and DCs that retained CD80 expression, instead upregulated PD-L1. Activated T cells regulate other cells by, e.g., IFN-γ [37]. Addition of recombinant IFN-γ (rIFN-γ) to the cell suspensions, upregulated CD80 on colonic MPs, but did not reduce CD80 levels on intratumoral MPs (Figure 6B). The CD80 expression on DCs was also not significantly altered by rIFN-γ. In line with previous murine studies [38], rIFN-γ upregulated PD-L1 on APCs (Figure 6B). 

Relative to the unstimulated control, pro-inflammatory and inhibitory cytokines were increased in the supernatants of microbeads cultures (Figure 6C). Increased levels of IL-1β, IL-6 and tumor necrosis factor (TNF) are indicative of APC activation and confirm the ability of APCs to respond to activated T cells. Although microbeads-stimulated colon suspensions had higher IFN-γ levels (Figure 6C), the colonic APCs in these suspensions were not more efficient in PD-L1 upregulation than intratumoral APCs (Figure 6A). There was a positive correlation of CD80 and PD-L1 on APCs in tumor and colon (Figure 6D), which suggests a negative feedback loop where CD80+ APCs that responded to activated T cells upregulate PD-L1 to reduce stimulatory cues from T cells. Substituting 10% of the culture media with supernatants from microbeads-stimulated cultures resulted in a similar correlated expression of CD80 and PD-L1 on autologous APCs in PBMC cultures (Figure 6E). 

Overall, induced T cell activation in cell suspensions from colon or tumor did not result in distinct patterns of CD80 and PD-L1 expression on co-residing APCs. Thus, in the scenario of CRC treatment-induced T cell activation, APCs in target and off-target tissues are likely comparably responsive to signals from activated T cells.

## 3. Discussion

Local immune responses in the distinct environment of tumor and adjacent colon, which stem from the interactions between infiltrating and/or tissue-residing T cells and APCs are largely unknown. Here, we characterized multiple intratumoral and colonic APC and T cell subsets from non-treated CRC patients, with regards to their ex vivo frequencies, phenotypic activation, and functional status. In addition, we attempted modelling of T cell activation after parenterally administrated CRC treatments, capable of direct or indirect targeting of T cells irrespective of their specificity, or anatomical location. Tissue-specific expression pattern of T cell co-stimulatory CD80 and T cell co-inhibitory PD-L1 were observed, especially on the MPs. Higher PD-L1 and lower CD80 expression on colonic APCs may imply greater propensity towards inhibitory responses compared to intratumoral APCs with opposed levels of these markers. It is possible that commensal microbes specifically in the colon, render colonic MPs more resistant to induce T cell activation. On the other hand, the abundance of intratumoral CD80+ APCs might represent a persistent tumor infiltration of circulating APCs that upregulate CD80 as they enter the non-sterile tumor environment. 

MSI-H tumors are considered more immunologically active than MSS tumors [32,33], which would impact infiltration of APCs. Correlation analyses implied DC infiltration was somewhat linked to homing of T cells to MSI-H tumors but, overall, the association between APC and T cell frequencies displayed a negative trend regardless of MSI status. Further, we did not observe significant differences in TIL numbers at the center or stroma of MSI-H vs. MSS tumors, which likely reflect the limited number of patients in the initial cohort for in situ analyses. These in situ observations and the predominance of MSS tumors in subsequent cohorts did not prompt further comparisons of TIL responses based on MSI status. Instead, we detailed the T cells infiltrating the tumor and adjacent colon by their expression of CD103 and CD39. These markers have been postulated to identify rare tumor-specific CD103+CD39+CD8+ TILs in solid tumors, including CRC [6,7]. 

Although the frequency of CD8 TILs co-expressing CD103 and CD39 was not increased relative to adjacent colon, these CD8 TILs had also significantly higher PD-1 and lower CD69 expression. Of note, CD4 and CD8 TILs with elevated PD-1 and decreased CD69 expression also co-expressed CD103, commonly used in the identification of TRMs. As high CD103+CD8+ TIL levels are linked with improved CRC patient survival [39], our analyses suggest that colonic and intratumoral CD103+CD8+ T cells with prominent PD-1 expression would be frequently targeted by anti-PD-1 ICI. Further, the relationship between the ex vivo activation status of APCs and T cells was observed on specific cell subsets and tissue. For example, activated CDC1 in tumor, but not colon, seem to confer CD69 downregulation on co-residing CD8+CD103+ TRMs. As CD69 identifies recently activated T cells but also tissue residency, it remains unclear if CD69 downregulation on CD103+ TRMs represent initial steps towards exiting the tissue, or potential sign of stimulation beyond the time frame for early activation.

Having completed the overview of the ex vivo phenotype and activation status of APC and TIL subsets, we proceeded to determine their predisposition to perform some of the essential immune functions. As protein processing by APCs precedes antigen presentation and stimulation of cognate T cells, we assessed uptake and degradation of protein by MPs and DCs. We have used protein labeled with AF647 dye, which remains detectable in intracellular acidic compartments containing degraded proteins. The higher frequencies of OVA-AF647+ colonic MPs and DCs would represent more efficient uptake, as well as degradation of the scarce amount of protein compared to corresponding subsets in the tumor. Further, evaluation of protein degradation alone via DQ-OVA showed that both colonic and intratumoral APCs were equally efficient. However, while colonic APCs that degraded protein significantly upregulated both CD80 and PD-L1, the intratumoral counterpart was more prone to only upregulate PD-L1. Whether upregulation of both CD80 and PD-L1 on colonic APCs during protein degradation would simultaneously convey contradicting signals to T cells during antigen presentation is unclear. As the intestine contains large numbers of memory T cells, it might be reasonable that CD80 and PD-L1 co-expression inhibit unjustified activation of tissue-residing T cells while the APCs process antigens. Relative to the tumor of CRC patients, higher PD-L1 expression on histological sections of the adjacent colon has also been reported earlier [31].

Treatment-induced modulations of immune cells likely shape the potency of anti-tumor immunity, severity of immune-related adverse events and overall survival of patients with CRC. Although, mouse models of CRC treatments have demonstrated the central role of APCs for induction and maintenance of T cell-mediated tumor rejection [24,25], the precise effects chemoradiation or ICI exert on the functional capacity of APCs and T cells located in target (tumor) and off-target (e.g., adjacent colon) tissues remain unclear. Therefore, we assessed the functional capacity of APCs subsequent to induced polyclonal T cell activation, since effector molecules from activated TILs would, in turn, uphold or limit APC functions during generation of anti-tumor immunity. Our attempts to simulate CRC treatment-induced global activation of T cells by stimulating T cells with microbeads, supported concurrent upregulation of CD80 and PD-L1 on APCs, regardless of whether they were from colon, tumor or blood. As IFN-γ induces PD-L1 on APCs [38], it is likely that CD80+ APCs would eventually also co-express PD-L1. Despite significantly higher IFN-γ concentration in stimulated colon suspensions, the colonic and intratumoral APCs were both efficient in upregulation of CD80 and PD-L1 in the presence of induced T cell activation. 

To this end, the tissue cell suspensions used in the study also contain other cells that may augment or interfere the functional responses of APCs. Highly purified APC and TIL subsets would be ideal for our in vitro assays, but the cell yields from available tissues were insufficient for in vitro assessments with purified cells. To simultaneously address whether tumor cells, stromal cells, granulocytes, myeloid-derived suppressor cells (MDSCs) or other lymphocytes, influence APC’s responses to induced T cell activation would require systems biological assessment of, e.g., the transcriptome of tumor vs. colonic tissues. Further, the current study is based on untreated CRC patients and whether intratumoral and colonic APCs respond differently to activated T cells after exposure to ICI or chemoradiation remains undetermined. In most cases, colonic or rectal resection is not considered for recently treated patients according to clinical guidelines, which greatly restricts the availability of these patient materials for study purposes. However, a few punch biopsies collected during routine post-treatment examination may suffice for a limited number of in vitro assays with bulk cell suspension.

## 4. Materials and Methods

### 4.1. CRC Patients

The patients were divided into three cohorts (n = 28, 10, and 17, respectively) based on the experimental procedure applied to collected samples. Colonic or rectal resection was performed at the Sahlgrenska University Hospital and additional patient data are provided in Supplementary Table S1. The patients had not been treated with immune-modulatory drugs or (chemo)radiation prior surgery. After collection of tumor tissue from the resectate, macroscopically tumor-free tissues were collected approximately 10 cm away from the tumor. Biopsies from tumor and colon were snapfrozen in liquid nitrogen for MSI analysis, or embedded in OCT (Histolabs, Gothenburg, Sweden) before sequential freezing in isopentane and liquid nitrogen for immunohistochemistry. Remaining tissues were transported in PBS on ice for generation of single-cell suspensions within one hour. Venous blood was collected into heparin tubes (BD, San Jose, CA, USA) during surgery. Tumor stages were specified in the pathology report on tumor invasion (T1-T4), lymph node involvement (N0-N2) and distant metastases (M0-M1). An overall stage (I-IV), based on TNM status, was subsequently determined. Microsatellite instability was analyzed by MSI Analysis System, version 1.2. (Promega, Nacka, Sweden) via fluorescently-labeled primers for co-amplification of mononucleotide repeat markers (BAT-25, BAT-26, NR-21, NR-24 and MONO-27) and pentanucleotide repeat markers (Penta C and Penta D). MSI of tumors were defined by peak alterations in the marker electropherogram relative to tumor-free control tissue. While instability in >1 mononucleotide repeat markers indicate MSI-H tumors, instability in 1 of these markers is considered MSI-L. Absence of MSI represents MSS tumors.

### 4.2. Immunohistochemistry

OCT embedded tissues stored in ^–^80 °C were placed inside –20 °C cryostat for 20 min prior sectioning. The 7 μm thick sections were fixed with 2% paraformaldehyde for 10 min, washed with PBS for 5 min, permeabilized with 0.1% Triton-X 100 (Sigma, St. Louis, MO, USA) and washed once more. Endogenous avidin and biotin were sequentially blocked for 10 min, respectively using Avidin/Biotin blocking kit (Biocare Medical, Pacheco, CA, USA) with PBS wash between and after these blocking steps. Sections were then incubated for 1 h at room temperature with a mixture of monoclonal antibodies diluted in PBS with 0.1% BSA (Sigma); anti- CD8 (clone RPA-T8), CD64 (clone 10.1), CD163 (clone RM3/1), EpCAM (clone G8.8) (Biolegend, San Diego, CA, USA), CD3 (clone UCHT1, BD) and CD11c (clone EP1347Y, Abcam, Cambridge, UK). All antibodies, except rabbit-anti CD11c, are fluorescence-conjugated and raised in mice. Thus, stained sections were incubated for 40 min with fluorescence-conjugated donkey anti-rabbit IgG (Jackson ImmunoResearch, West Grove, PA, USA). Finally, sections were mounted with Prolong Diamond Antifade with DAPI (Invitrogen, Thermo Fisher, Carlsbad, CA, USA). Sections were scanned with Metafer Slide Scanning Platform (Metasystems, Heidelberg Germany) with Axio Imager.Z2 Microscope, 20×/0.8/air objective (Zeiss, Oberkochen, Germany) and SpectraSplit filter set (Kromnigon, Gothenburg, Sweden). TILs and APCs were quantified within 1 mm^2^ region by Fiji/ImageJ software with Cell Counter plugin. For simplification, the sole MSI-L tumor was allocated to the MSS tumor group for immunohistochemistry.

### 4.3. Generation of Single Cell Suspensions

Cell suspensions of tumor and adjacent control tissue were generated as described [37]. Briefly, 3 × 3 mm tissue pieces were washed in HBSS without Mg^++^/Ca^++^ (Gibco, Thermo Fisher Carlsbad, CA, USA) supplemented with 2% FBS (Gibco), 1% Hepes buffer and 2 mM EDTA (Lonza, Basel, Switzerland). Tissues were digested with 70 μg/mL Liberase TM (Roche, Basel, Switzerland) and 20 μg/mL DNase I (Sigma) in complete media consisting of RPMI 1640 with 10% FCS, 1% Penicillin/Streptomycin, 1% Hepes buffer and 0.1% Gentamycin (Gibco). Cell suspensions were filtered, washed and resuspended to 1 × 10^6^ cells/mL. PBMCs were isolated via Ficoll gradient (GE Healthcare, Uppsala, Sweden).

### 4.4. OVA Uptake and Degradation

For evaluation of uptake and degradation, tumor and colon tissue suspensions were cultured overnight in presence of 0.4 µg AlexaFluor 647 (AF647)-conjugated OVA (Invitrogen). To solely assess degradation, 0.6 μg DQ-OVA (Invitrogen) was used instead. Efficiency of uptake and degradation of OVA were analyzed with flow cytometry. 

### 4.5. Stimulation of APCs by Activated T Cells

Single cell suspensions from tumor and colon tissues were resuspended in complete medium to 1 million cells/mL in 5-mL polystyrene tubes (Corning, Amsterdam, Netherlands). Stimulation of tissue APCs by polyclonally activated co-residing T cells was performed by addition of 2.5 µL microbeads loaded with anti-CD2, CD3 and CD28 antibodies (Miltenyi Biotec, Auburn, CA, USA), which represent 1 bead: 4 cell ratio. In parallel, cell suspensions were stimulated with 0.2 μg/mL recombinant IFN-γ (Biolegend, San Diego, CA, USA). After 6 h of culture, supernatants from unstimulated and microbeads-stimulated cell suspensions were collected from the top layer of the culture medium to avoid contamination of sedimented cells or microbeads. Moreover, autologous PBMCs were also cultured for 12 h in 90% complete media and 10% supernatant from microbeads-stimulated tissue suspensions. Remaining supernatants were stored at −20 °C until use. Activation of APCs was analyzed by flow cytometry. 

### 4.6. Supernatant Cytokine Analyses

Cytokines (IFN-γ, IL-1β, IL-6, IL-10, IL-17A, IL-21, IL-22 and TNF) in supernatants from microbeads-stimulated tumor and control tissue suspensions were analyzed by Meso Scale discovery (MSD) multiplex platform using the U-PLEX TH17 Combo2 kit (Meso Scale Diagnostics, Rockville, MD, USA), according to manufacturer’s protocol. 

### 4.7. Flow Cytometry 

Cell suspensions were stained, as described [37,40]. Briefly, cell viability in 1 mL cell suspension containing 1 million cells was determined by Zombie Red Fixable viability dye (Biolegend), followed by 20 min incubation with fluorescence-conjugated antibodies (Supplementary Table S2). Stained cells were fixed with 2% paraformaldehyde. AccuCount beads (Spherotech, Lake Forest, IL, USA) were used for cell enumeration, according to manufacturer’s protocol. Samples were acquired using BD LSRFortessa flow cytometer and analyzed with FlowJo v.9.9.6 (Tree Star, Ashland, OR, USA).

### 4.8. Statistical Analysis

Paired and unpaired comparisons were made using Wilcoxon signed-rank test and Mann–Whitney test, respectively. Correlation matrixes and bubble plots were based on non-parametric Spearman correlation analyses. Where indicated, linear regression was applied instead. All analyses were performed using GraphPad Prism software, v.9.0.2. (San Diego, CA, USA) and considered significant at *p* < 0.05.

## 5. Conclusions

In conclusion, our extensive characterization of APCs and T cells from the contrasting environments of tumor and colon of patients with CRC enabled determination of tissue-specific activation status and functional capacity of specific APC and T cells. The distinct milieus of tumor and colon are likely reflected on the opposed expression of CD80 and PD-L1 on MPs, as well as PD-1 and CD69 on CD8+CD103+CD39+ T cells. The colonic APCs’ superior protein uptake and subsequent upregulation of CD80 and PD-L1 compared to tumor APCs, plus the enhanced IFN-γ responses of colonic T cells might indicate different functional propensity fostered in tumor vs. colon. Overall, colonic or intratumoral APCs displayed similar capacity to respond to activated T cell-mediated regulation of their co-stimulatory CD80 and co-inhibitory PD-L1 expression. The presented data could provide insights into tissue APCs’ post-treatment induction and/or maintenance of local anti-tumor responses or immune-related adverse events affecting the normal colon.

## Data Availability

Data is contained within the article and supplementary material.

## Supplementary Materials

The following are available online at https://www.mdpi.com/article/10.3390/cancers13205247/s1, Figure S1: Associations of APCs and TILs according to tumor stage and MSI status. Figure S2: Spearman correlation coefficients and p-values from association analyses of APC and T cell subsets. Table S1: Characteristics of colorectal cancer patients, Table S2: Fluorescence-conjugated antibodies for flow cytometry.
