# Supplementary material for: Antigen Presenting Cells from Tumor and Colon of Colorectal Cancer Patients Are Distinct in Activation and Functional Status, but Comparably Responsive to Activated T Cells
Source: Cancers (Basel). 2021 Oct 19;13(20):5247. doi: 10.3390/cancers13205247 (PMC8533845; doi:10.3390/cancers13205247)
Supplement: Supplementary file 1 [file cancers-13-05247-s001.zip › cancers-1411321-supplementary.pdf]

Supplementary Materials

Table S1. Characteristics of colorectal cancer patients.

		**Females**	**Males**
	n (55)	23	32
	Age range	69-88	37-90
	Age median	77	73
Tumor location	Right colon	18	14
	Left colon	5	13
	Rectum	0	5
TNM stage	I	4	7
	II	9	11
	III	9	13
	IV	1	1
Microsatellite stability	MSS	12	27
	MSI-H	11	3
	MSI-L	0	1
	Unknown	0	1

Table S2. Fluorescence-conjugated antibodies for flow cytometry.

Antibody	Clone	Manufacturer
CD1c	L161	Biolegend
CD3	UCHT1	BD Biosciences
CD4	OKT4	Biolegend
CD8	RPA-T8	BD Biosciences
CD11c	B-ly6	BD Biosciences
CD14	M5E2	Biolegend
CD15	HI98	BD Biosciences
CD16	3G8	Biolegend
CD19	SJ25C1	BD Biosciences
CD39	A1	Biolegend
CD45	HI30	BD Biosciences
CD56	HCD56	Biolegend
CD64	10.1	BD Biosciences
CD69	FN50	BD Biosciences
CD80	L307.4	BD Biosciences
CD86	FUN-1	BD Biosciences
CD103	Ber-ACT8	BD Biosciences
CD141	1A4	BD Biosciences
HLA-DR	G46-6	BD Biosciences
PD-1	EH12.1	BD Biosciences
PDL-1	29E.2A3	Biolegend

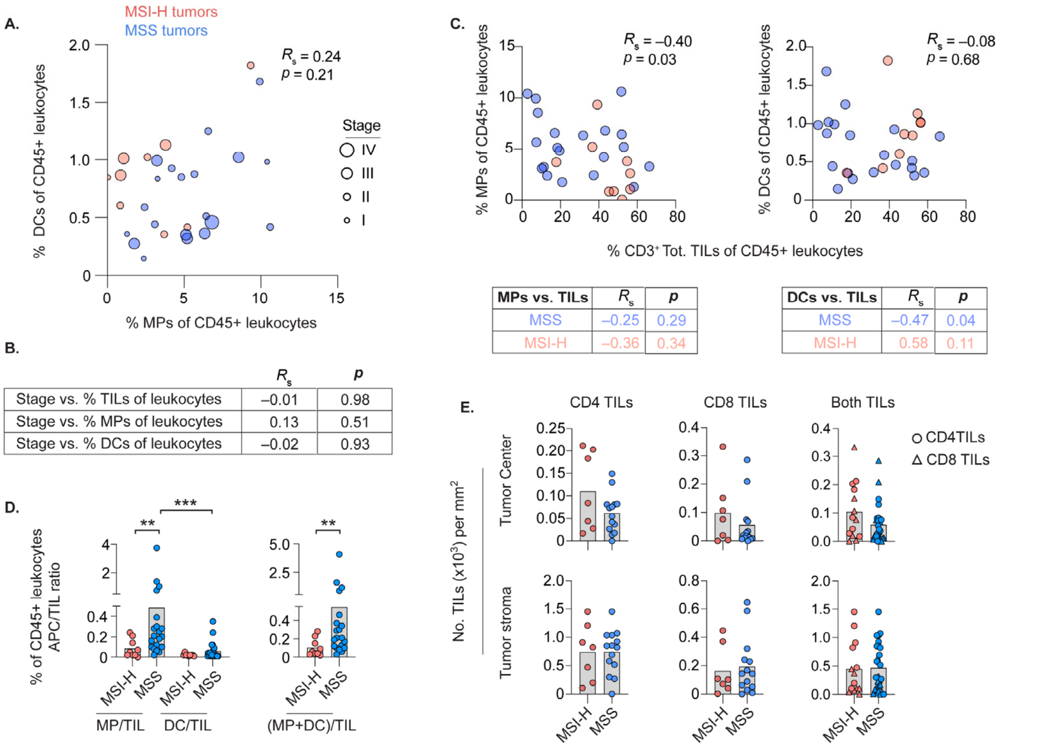

Figure S1. Associations of APCs and TILs according to tumor stage and MSI status. (**A**) Bubble plot with Spearman correlation analysis of the percentages of intratumoral CD64+ MPs vs. CD64- DCs within viable CD45+ leukocytes. Each circle represents an individual patient with CRC. The size and color of the circles correspond to specific tumor stage and MSI status, respectively. (**B**) Table with Spearman R_s values and p-values from association analyses of tumor stage vs. frequencies of TILs, MPs and DCs, respectively. (**C**) Spearman correlation analysis of the percentages of CD3+ TILs vs. MPs or DCs within leukocytes isolated from MSS (blue) and MSI (red) tumors. Tables below show the Spearman R_s values and corresponding p-values for analysis on either MSS or MSI-H tumors alone. (**D**) Ratio of indicated APC subset to TILs according to their respective percentages among leukocytes in MSS and MSI-H tumor suspensions. (**E**) Number of indicated TILs per mm^2 in the tumor center and stroma of MSI-H and MSS tumor sections. Bars show the mean. (**$p < 0.01$, ***$p < 0.001$, Mann-Whitney test).

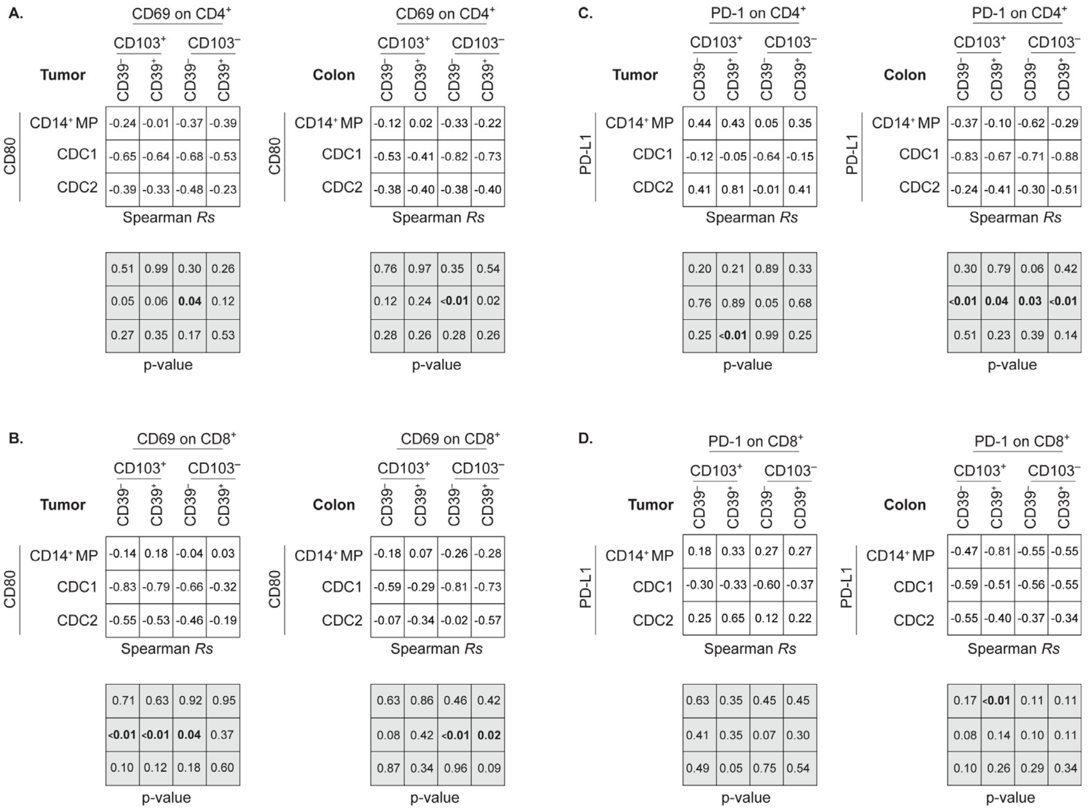

Figure S2. Spearman correlation coefficients and p-values from association analyses of APC and T cell subsets. Spearman correlation between CD80 MFI of APC subsets vs. CD69 MFI of CD4 (A), or CD8 (B) T cell subsets in tumor and adjacent colon. Similar analyses on PD-L1 MFI of APC subsets vs. PD-1 MFI on CD4+ (C), or CD8+ (D) T cell subsets in tumor and colon. Top matrixes show Spearman correlation coefficient *Rs* values and grey matrixes indicate the corresponding p-values. ($p < 0.05$ and $p<0.01$ are indicated in bold).
